# Intraoperative Electroretinograms before and after Core Vitrectomy

**DOI:** 10.1371/journal.pone.0152052

**Published:** 2016-03-24

**Authors:** Kazuma Yagura, Kei Shinoda, Soiti Matsumoto, Gaku Terauchi, Emiko Watanabe, Harue Matsumoto, Goichi Akiyama, Atsushi Mizota, Yozo Miyake

**Affiliations:** 1 Department of Ophthalmology, Teikyo University School of Medicine, Itabashi-ku, Tokyo, Japan; 2 Matsumoto Eye Clinic, Takagaki, Awa-cho, Awa-shi, Tokushima, Japan; 3 Akiyama Eye Clinic, Kita-ku, Tokyo, Japan; 4 Aichi Medical University, Yazakokarimata, Nagakute-shi, Aichi, Japan; Univeristy of Miami, UNITED STATES

## Abstract

**Purpose:**

To evaluate retinal function by intraoperative electroretinograms (ERGs) before and after core vitrectomy.

**Design:**

Retrospective consecutive case series.

**Method:**

Full-field photopic ERGs were recorded prior to the beginning and just after core vitrectomy using a sterilized contact lens electrode in 20 eyes that underwent non-complicated vitreous surgery. A light-emitted diode was embedded into the contact lens, and a stimulus of 150 ms on and 350 ms off at 2 Hz was delivered. The amplitudes and latencies of the a-, b-, and d-waves, photopic negative response (PhNR), and oscillatory potentials (OPs) were analyzed. The intraocular temperature at the mid-vitreous was measured at the beginning and just after the surgery with a thermoprobe.

**Results:**

The intraocular temperature was 33.2 ± 1.3°C before and 29.4 ± 1.7°C after the vitrectomy. The amplitudes of the PhNR and OPs were significantly smaller after surgery, and the latencies of all components were prolonged after the surgery. These changes were not significantly correlated with the changes of the temperature.

**Conclusion:**

Retinal function is reduced just after core vitrectomy in conjunction with significant temperature reduction. The differences in the degree of alterations of each ERG component suggests different sensitivity of each type of retinal neuron.

## Introduction

Vitreous temperature has been reported to fluctuate during vitreous surgery.[1–3] Thus, Landers et al^1^ measured the temperature of the retinal surface during vitreous surgery with a 23-gauge flexible wire thermoprobe in 7 cases. They found that the retinal surface temperature decreased during vitrectomy and recovered 5 minutes after the completion of the vitrectomy. Miyake and colleagues[4–6] recorded intraoperative electroretinograms (ERGs) and reported a reduction in the amplitude and prolongation of the latency of the 30 Hz flicker ERGs. They also showed that the change depended on the temperature of the irrigating fluid.[5,6] However, a layer-by-layer analysis of the retinal function was not done and *in situ* measurements of the intraocular temperature was not made.

The purpose of this study was to evaluate retinal function by intraoperative ERGs before and after core vitrectomy. To accomplish this, we recorded photopic ERGs and measured the temperature in the vitreous cavity before and after core vitrectomy. Because the photopic ERGs allow layer-by-layer analyses of retinal function, we were able to evaluate the functional changes in each retinal layer after the core vitrectomy.

## Methods

### Patients

The participants were scheduled to undergo vitreous surgery in 2014 at the Teikyo University Hospital in Tokyo, Japan for various reasons. All patients had given consent for the operation with intraoperative ERG recordings and temperature measurements. Of the 20 eyes of 20 patients, 14 eyes of 14 men and 6 eyes of 6 women. The average age of the patients was 71.5 ± 8.2 (±SD) years with a range from 58 to 86 years. The vitreoretinal pathologies were; 6 with a macular hole, 1 with macular edema, 9 with an epiretinal membrane, 2 with macular telangiectasia, and 2 with an intraocular lens (IOL) dislocation. The protocol for this study was approved by the Institutional Review Board of Teikyo University, and a written informed consent was obtained from all of the patients.

### Methods

**Fig 1:**
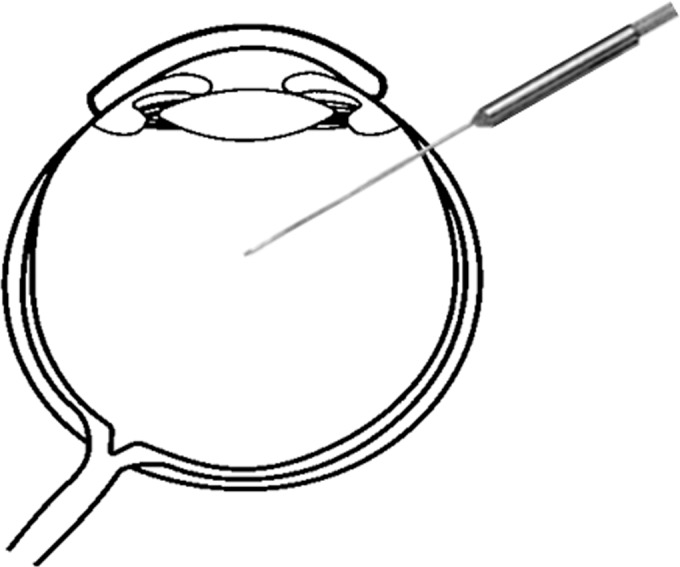
Appearance of the eye during intravitreal temperature recording. The tip of a 26-gauge flexible wire thermoprobe (MT-26, Phisitemp, New Jersey USA) was inserted through trocar and placed in the midvitreous cavity. The thermoprobe was connected to a battery-operated thermometer device (Phisitemp BAT-12, New Jersey USA).

Temperature measurements were made with a 26-gauge flexible wire thermoprobe (MT-26, Phisitemp, New Jersey USA, **Fig 1**). The thermoprobe is accurate within ± 0.1°C, equilibrates with the surrounding fluid within 2 to 3 sec, and measures temperature only at the very tip of the device. The thermoprobe was sterilized in ethylene oxide gas. Intraoperatively, the tip was inserted through a trocar and located in the mid vitreous cavity with the position detected by a wide viewing system and operating microscope. The thermoprobe was connected to a battery-operated thermometer device (Phisitemp BAT-12, New Jersey, USA).

The CONSTELLATION^®^ Vision System (Alcon, Fort Worth, TX) with 25-gauge instruments was used for the vitrectomy. All surgeries were performed under sub-Tenon anesthesia. Patients were prepped and draped in the usual sterile fashion, and valved trochars were placed at the conventional sites. When necessary, standard phacoemulsification and intraocular lens implantation with corneal incision was done prior to the 25-gauge vitrectomy. The temperature measurements were made just before and just after the core vitrectomy. The room temperature was set at 25°C. The thermoprobe was removed from sterile packaging and connected to the digital thermometer device. The tip of the thermoprobe was passed through a trocar into the vitreous cavity, and the location of the tip was viewed with a binocular indirect ophthalmoscope (OCULUS Optikgeräte GmbH, Wetzlar, Germany). The intravitreal temperature measurements were taken by placing the tip of the thermoprobe in the midvitreous cavity. The total time required to take these measurements was approximately 1 minute. The thermoprobe was removed and full-field ERG recordings were made. After the ERG recordings, core vitrectomy was done followed by mid vitreous temperature measurement and a second ERG. To minimize variability in the location of temperature measurements, one of the authors was present during the temperature measurements and intraoperative electroretinographic (iERG) recordings.

### Intraoperative electroretinograms (iERGs)

**Fig 2:**
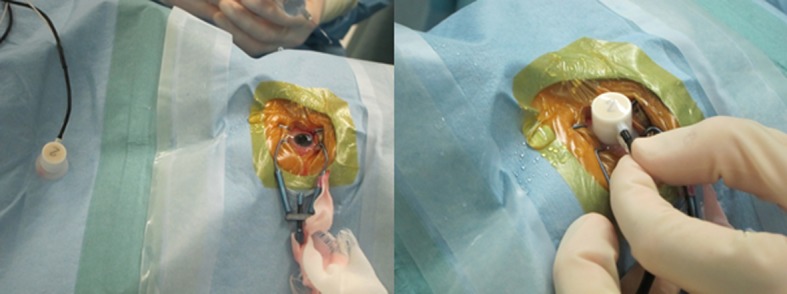
Appearance of an eye during intraoperative electroretinographic recordings. A contact lens with a built-in light-emitting diode (LS-100, Mayo Co, Inazawa, Japan) was sterilized and used as both the stimulus source and recording electrode for photopic ERGs.

A contact lens with a built-in light-emitting diode (LS-100, Mayo Co, Inazawa, Japan) was sterilized and used as both a stimulus source and a recording electrode for the photopic iERGs **(Fig 2)**. The reference electrode was a silver plate placed on the forehead and the ground electrode was attached to one ear lobe. The photopic ERGs were elicited by 2-Hz rectangular stimuli with 150-ms light on and 350-ms light off and a luminance of 398.1 cd/m^2^. The luminance of the constant background was 39.8 cd/m^2^.

**Fig 3:**
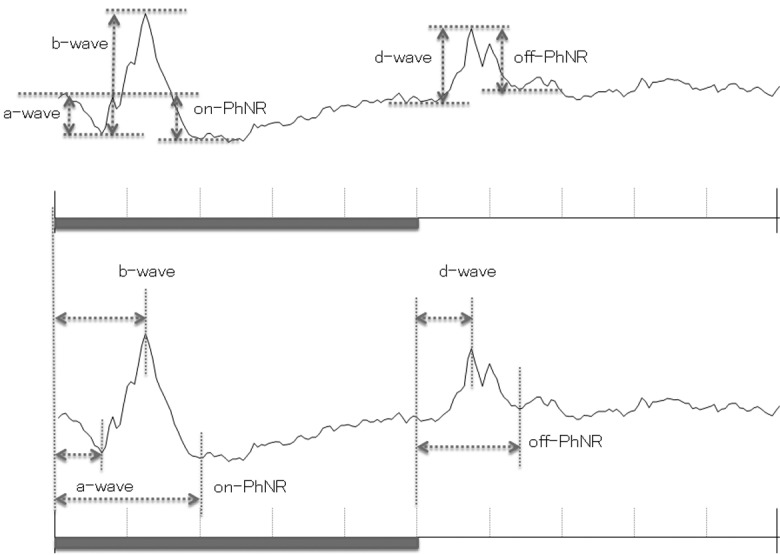
Measurements of each component. The amplitude (upper) and latency (lower) of each component are shown. bar represents the beginning and duration of the stimulus flash.

**Fig 4:**
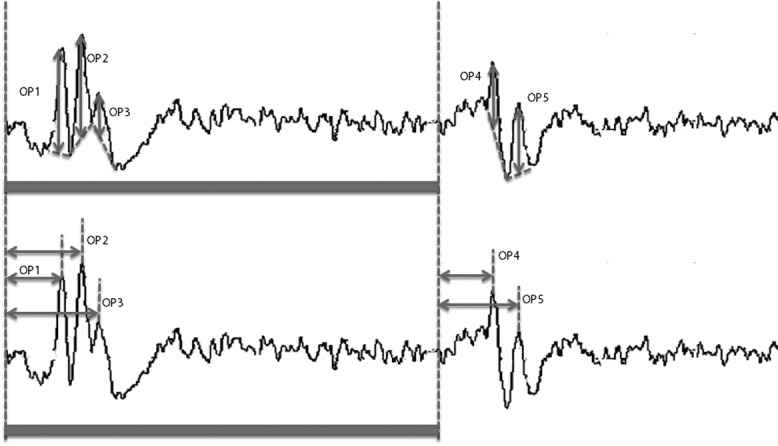
Measurements of the amplitude and the latency of the oscillatory potentials (OPs). The amplitude (upper) and latency (lower) of each OP are shown. bar represents the beginning and duration of the stimulus flash.

The iERGs were amplified and averaged with a bioamplifier (MEB-9404, Nihon Kohden Corporation, Tokyo, Japan), and A/D converted to 16 bits (PCI-16/16UD, Contec, Japan). Twenty responses were averaged, and the sampling rate was 10 kHz. The responses were filtered to 2 Hz to 1 kHz with a hardwired band pass filter for recording the a-, b-, and d-waves and to 100 Hz to 500 Hz to record the oscillatory potentials (OPs). The recording window was 300 ms. All ERG recordings were performed after 5 minutes of room light-adaptation. The amplitudes and latencies of the b-, and d-waves, and photopic negative responses (PhNRs) following the b-wave, and the d-waves (on-PhNR and off-PhNR, respectively), and OPs were analyzed. Briefly, the latencies of the a-, b- and d-waves, the PhNR, and the OPs were measured from stimulus onset to the peak of each wave **(Figs 3 and 4)**. The amplitudes of the a-waves were measured from baseline to the troughs of the a-waves, and the amplitudes of the b-wave were measured from the troughs of the a-waves to the peaks of the b-waves, and the amplitudes of the PhNR were measured from the baseline to the peak troughs of the on- or off-PhNR, respectively **(Fig 3)**. The amplitudes of the OPs were measured from the baseline, which was determined as the line connecting each negative trough, to the peak **(Fig 4)**.

### Statistical analyses

The significance of differences in the temperatures, amplitudes, and latencies of the a-, b-, and d-waves, on-PhNR, off-PhNR, and OPs, before and after core vitrectomy was determined by paired *t* tests. Pearson’s correlation coefficient was used to determine the significance of the correlations between the change of temperature and each ERG parameter. A *P* <0.05 was taken to be statistically significant.

## Results

**Fig 5:**
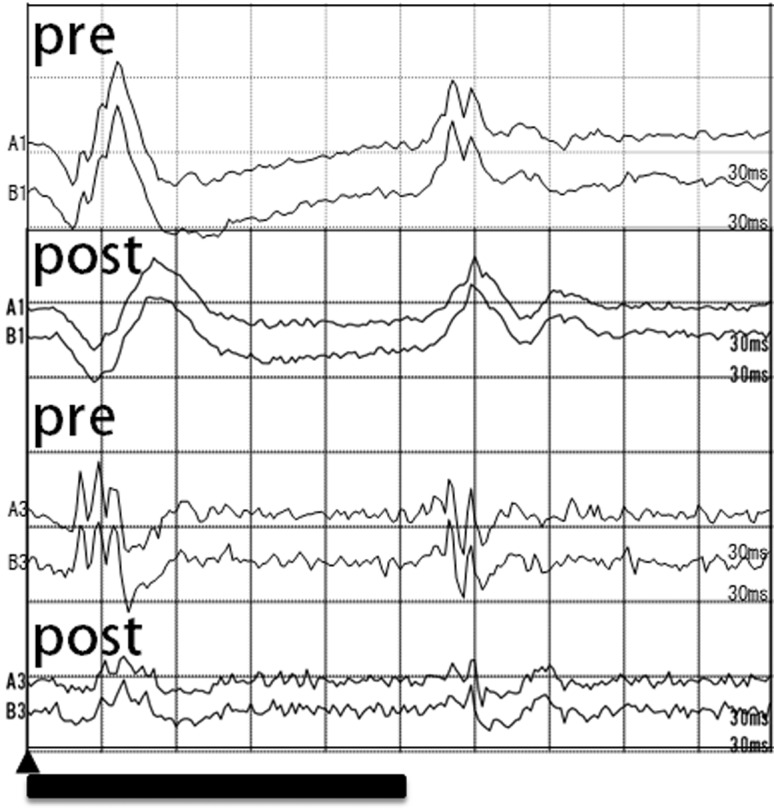
Representative ERGs and temperatures before and after core vitrectomy. The intravitreal temperature was 35.2°C before and 33.9°C after core vitrectomy.▲ and bar represents the beginning and duration of the stimulus flash.

**Fig 6:**
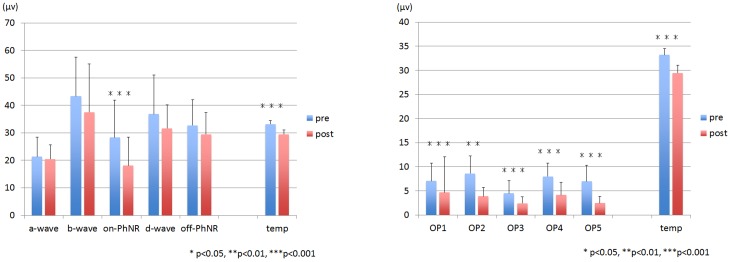
The amplitude of each component and the intravitreal temperature before and after core vitrectomy. The amplitude of the on-PhNR, OP2, OP3, OP4, and OP5 decreases significantly after core vitrectomy. PhNR: photopic negative response, OP: oscillatory potential, bar indicates standard error. *P <0.05, **P <0.01, ***P <0.001.

**Fig 7:**
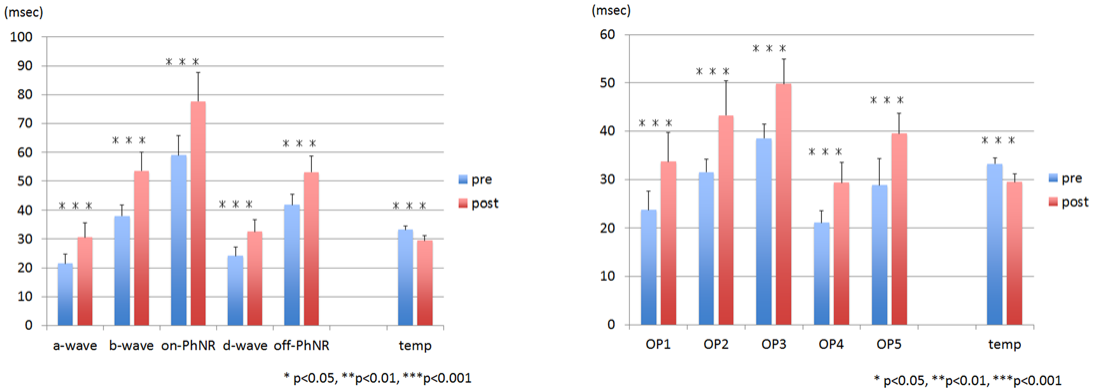
The latency of each component and the intravitreal temperature before and after core vitrectomy. The latency of each component is significantly delayed after core vitrectomy. PhNR: photopic negative response, OP: oscillatory potential, bar indicates standard error. *P <0.05, **P <0.01, ***P <0.001

The mean amplitudes of the post-vitrectomy on-PhNR, OP2, OP3, OP4, and OP5 were significantly smaller than that of the corresponding pre-vitrectomy responses (**Figs 5, 6A and 6B, Table 1**). The mean post-vitrectomy latency of each component was significantly longer than that of the pre-vitrectomy latency (**Figs 5, 7A and 7B, Table 1**).

**Table 1:** Changes of the electrophisiologic parameters and the intravitreal temparature before and after core vitrectomy.

**amplitude(uV)**										
	**a-wave**	**p value**	**b-wave**	**p value**	**on-PhNR**	**p value**	**d-wave**	**p value**	**off-PhNR**	**p value**
**pre**	**21.35±7.08**	**0.48470677**	**43.35±14.14**	**0.11985184**	**28.3±13.58**	***0*.*00017395***	**36.8±14.21**	**0.07105886**	**32.75±9.37**	***0*.*26110339***
**post**	**20.5±5.09**		**37.55±17.58**		**18.1±10.25**		**31.65±8.62**		**29.5±7.83**	
**latency(msec)**										
	**a-wave**	**p value**	**b-wave**	**p value**	**on-PhNR**	**p value**	**d-wave**	**p value**	**off-PhNR**	**p value**
**pre**	**21.45±3.27**	***2*.*47E-08***	**37.95±3.93**	***5*.*27E-11***	**58.95±6.9**	***5*.*9241E-10***	**24.15±3.0**	***2*.*5221E-07***	**41.85±3.44**	***2*.*72E-08***
**post**	**30.6±4.92**		**53.55±6.33**		**77.7±9.92**		**32.55±4.16**		**53.1±5.51**	
**amplitude(uV)**										
	**OP1**	**p value**	**OP2**	**p value**	**OP3**	**p value**	**OP4**	**p value**	**OP5**	**p value**
**pre**	**7.07±3.65**	**0.17859439**	**8.6±3.62**	***4*.*0256E-06***	**4.47±2.69**	***0*.*00639687***	**8±2.76**	***5*.*7025E-05***	**6.98±3.35**	***5*.*4395E-05***
**post**	**4.65±7.42**		**3.87±1.85**		**2.4±1.35**		**4.13±2.62**		**2.44±1.38**	
**latency(msec)**										
	**OP1**	**p value**	**OP2**	**p value**	**OP3**	**p value**	**OP4**	**p value**	**OP5**	**p value**
**pre**	**23.75±3.81**	***6*.*5995E-06***	**31.5±2.67**	***5*.*0376E-07***	**38.53±2.87**	***2*.*8302E-07***	**21.15±2.48**	***1*.*8191E-10***	**28.8±5.44**	***1*.*2112E-05***
**post**	**33.7±6.05**		**43.26±7.16**		**49.8±5.05**		**29.3±4.15**		**39.5±4.15**	
**temperature (degrees centigrade)**										
		**p value**								
**pre**	**33.2±1.28**	***4*.*1491E-08***								
**post**	**29.45±1.65**									

PhNR:photopic negative response

OP:oscillatory potential

p-value of significance is shown in italic.

**Fig 8:**
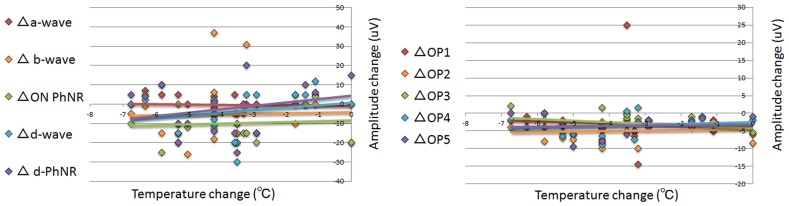
The relationship between the change in the amplitude of each component and in the intravitreal temperature before and after core vitrectomy. No significant correlation was found in each relationship; amplitude change vs temperature change. There was a tendency for the amplitude in the d-wave and the off-PhNR to decrease with decreasing the temperature. Δ: the difference between pre- and post core vitrectomy that was determined as the value of the post vitrectomy minus that of the pre vitrectomy. PhNR: photopic negative response, OP: oscillatory potential,

**Fig 9:**
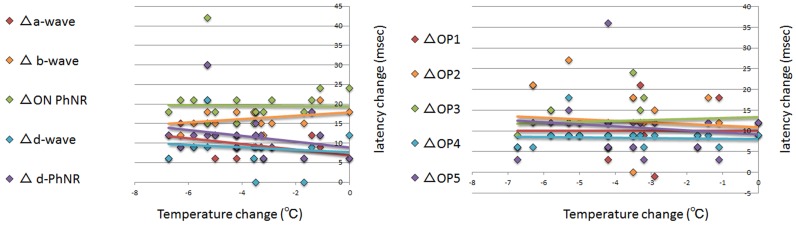
The relationship between the change in the latency of each component and in the intravitreal temperature before and after core vitrectomy. No significant correlation was found in each relationship; latency change vs temperature change. There was a tendency for the latency in the a- and d-waves and the off-PhNR to be prolonged and for the latency in the b-wave to be shortened with decreasing the temperature. Δ: the difference between pre- and post core vitrectomy that was determined as the value of the post vitrectomy minus that of the pre vitrectomy. PhNR: photopic negative response, OP: oscillatory potential.

The mean vitreous temperature was 33.2 ± 1.3°C before and 29.4 ± 1.7°C after the core vitrectomy (*P* <0.001, **Figs 6 and 7, Table 1**). Graphs of the change in amplitude and latency of each ERG component against temperature change are shown in **Figs 8 and 9**. The coefficients of correlation between the pre- and post-vitrectomy amplitudes of the different components and the vitreous temperatures were not significant. Similarly, the coefficients of correlation between the pre- and post-vitrectomy latencies and the vitreous temperature were not significant.

## Discussion

Our measurements showed that the midvitreous temperature was significantly lower just after core vitrectomy which is in good accordance with previous reports.^1-3^ Landers et al^1^ compared the intraocular temperatures at three time points; after preparation of sclerotomy sites but before opening the infusion line, at end of active vitrectomy with an open infusion line, and five minutes after closing the infusion line with the sclerotomies plugged. The mean midvitreous temperature was 33.9°C, 24.9°C, and 30.6°C, before, during, and after the vitrectomy, respectively, and the differences were statistically significant. Iguchi et al^3^ reported that the midvitreous temperature was 33.0°C before and 30.7°C after core vitrectomy which is in good agreement with our results in spite of several different surgical procedures.

It was expected that the temperature changes would have some effect on the retinal function. In the isolated rabbit retina, a shortening of the implicit time with an increase temperature have been reported.[7] In addition, an increase in the steepness and amplitude of the a-waves with increasing temperature (temperature range: 10 to 34°C.) have been reported.[8] Reduced ERG amplitudes and prolongation of the intrinsic times were shown in cat, guinea pig, and mouse eyes.[9–11] Tazawa et al [12] investigated the effect of the retinal temperature on retinal function by recording ERGs from isolated perfused extracorporeal bovine eyes under hypothermic condition. They reported a slight decrease of the amplitude and a prolongation of latencies of the a- and b-waves and a marked decrease in the amplitudes and latencies of the OPs when the temperature was reduced from 37°C to 30°C. They concluded that hypothermia affected the ERGs and affected the different components differently.

In human eyes, intraoperative recording of the 30 Hz flicker ERGs demonstrated an acute, reversible reduction in the amplitude and prolongation of the latency just after vitrectomy.[4–6] The changes were attributed to the lower temperature of the irrigating solution of 28°C instead of 35°C. The authors mentioned that hypothermia appeared to inhibit the viability and metabolic activity of the retinal cells and electrochemical events responsible for the electrical responses of the retina to light.

In our study, the change of the midvitreous temperature was approximately -3.0°C, and we recorded photopic iERGs with long duration stimuli. The recording of these iERGs allowed for a layer-by-layer analyses of the retinal function in each retinal layer during core vitrectomy. Because this difference was smaller than that in the earlier studies, and because the retinal temperature should be higher than that at the midvitreous, direct comparisons cannot be made. However, our results showed that routine vitreous surgery caused significant reductions of the PhNR and the OPs. Because the PhNRs and Ops arise from the neural activity of the inner and middle retinal layer, respectively, our results suggest that the decrease of vitreous temperature may have greater influence on the inner and middle retinal layers. Interestingly, the results showed an increase in timing of the a-wave and b-wave without a change in amplitude. Several investigators reported reduced amplitudes and prolongation of the implicit times with temperature decrease. [6,9,12,13] However, no information about which change was sensitive, i.e. reduction in amplitude or prolongation in implicit time, were provided. Wolin et al. [9] proposed that the changes in the latency are due to the effect of cold on the photochemical and neurochemical reactions at the reduced ambient temperature and that the reduced amplitudes may be partly a function of fewer receptors and/or nerve fibers of different calibers and myelination being excited at any given time.

The present study showed no significant correlation between ERG and mid vitreous temperature, although there was a tendency for the amplitude of some components to decrease with decreasing temperature. The ERG changes may also be related to other unidentified factors rather than just to temperature. Vitrectomy causes changes in electrolytes and osmolality as well as indirect mechanical changes to the retina, which may contribute to the ERG changes. [14–16] In addition, we measured the midvitreous temperature and not the retinal temperature. The retinal temperature is more likely to correlate with some of the ERG components than vitreal temperature. However, it is unlikely that retinal temperature is as much affected as vitreal temperature because of the retinal circulation.

A related intriguing and important question is whether the ERG returns to normal when the temperature does. However, we could not look into this question as the temperature did not return to normal during surgery. [3] In addition, further procedures could compromise the patients’ treatment and also influence the ERG.

Our study has several limitations such as the relatively small sample size and the variation in the vitreoretinal pathologies of the participants. Some ERG components may behave differently depending on the disease. For example, the OPs are attenuated in early stage of diabetic retinopathy [17] because the retinal ganglion cells are susceptible to ischemia,[18,19] and the PhNR may be more altered in eyes with retinal circulatory disorder. [20] A larger number of patients would enable subgroup analysis with each vitreoretinal disease and then ERG changes in specific vitreoretinal pathology could be determined.

Despite these limitations, we believe that the current data are clinically of great importance.

In conclusion, intraoperative photopic ERGs enabled monitoring of the dynamic changes of retinal function during surgery. Retinal function is reduced just after core vitrectomy in conjunction with midvitreous temperature reduction. The response of each ERG component varies suggesting different sensitivity of each type of retinal neuronal element.
